# Saliva Decreases Sucrose-Induced Cariogenicity in an Experimental Biological Caries Model

**DOI:** 10.3390/microorganisms11061426

**Published:** 2023-05-29

**Authors:** Rodrigo A. Giacaman, Rodrigo Umaña, María José Nuñez, Natalia Díaz-Garrido, Constanza Echeverría, Natalia García-Manríquez, Alex Mira, Constanza E. Fernández, Karla Gambetta-Tessini, Carla P. Lozano

**Affiliations:** 1 Cariology Unit, Department of Oral Rehabilitation, Faculty of Dentistry, University of Talca, Talca 3462227, Chile; rodrigoumanae@gmail.com (R.U.); mjosenunezg@gmail.com (M.J.N.); natalia.diaz.garrido@gmail.com (N.D.-G.); constanzaecheverriagarces@gmail.com (C.E.); nagarcia@utalca.cl (N.G.-M.); cofernandez@utalca.cl (C.E.F.); kgambetta@utalca.cl (K.G.-T.); 2 Interuniversity Center for Healthy Aging, Consortium of Chilean State Universities, Chilecito 03825, Chile; 3 Genomics and Health Department, Foundation for the Promotion of Health and Biomedical Research (FISABIO), 46020 Valencia, Spain; mira_ale@gva.es; 4 Laboratory of Biochemistry and Oral Biology, Institute for Research in Dental Sciences, Faculty of Dentistry, University of Chile, Santiago 8330111, Chile; clozano@odontologia.uchile.cl

**Keywords:** saliva, caries, biofilm, cariogenicity, *Streptococcus mutans*

## Abstract

Objective. Whether a minimum quantity of saliva inhibit the caries process remains uncertain. This study aimed to investigate the impact of saliva dilutions on an in vitro caries model using *Streptococcus mutans* (*S. mutans*) biofilms. Methods. *S. mutans* biofilms were cultivated on enamel and root dentin slabs, in culture media containing different proportions of saliva (*v*/*v*): 0%, 5%, 10%, 25%, 50%, 75%, and 100% saliva, and exposed to a 10% sucrose solution (5 min, 3x/day), with appropriate controls. After 5 (enamel) and 4 (dentin) days, demineralization, biomass, viable bacteria, and polysaccharide formation were analyzed. The acidogenicity of the spent media was monitored overtime. Each assay was performed in triplicate across two independent experiments (n = 6). Results. In both enamel and dentin, an inverse relationship was observed between acidogenicity, demineralization, and the proportion of saliva. Even small quantities of saliva incorporated into the media led to a noticeable reduction in enamel and dentin demineralization. Saliva presence resulted in significant reductions in biomass, viable *S. mutans* cells, and polysaccharides, with the effects being concentration-dependent for both tissues. Conclusions. High quantities of saliva can almost completely inhibit sucrose-induced cariogenicity, while even small amounts exhibit a dose-dependent caries-protective effect.

## 1. Introduction

Several substances with reported anticariogenic properties, such as statherin, proline-rich proteins, cystatins, and histatins, have been identified in saliva [1]. In addition, saliva contains several compounds with pH buffering capacity, that restore pH after a sugar-induced acidification event [2]. Thus, it has been assumed that a normal salivary flow would be protective against caries, while decreased flow would be related to higher caries prevalence [3]. Putative normal thresholds for salivary flow rate, pH, and buffer capacity have been widely accepted [4]. Variations in saliva flow and quality can be affected by several physiological or pathological factors, such as age, sex, medication, and systemic diseases, nonetheless [5]. Despite the wide consensus on the current conceptualization of dental caries [6], the role of saliva in caries development has received relatively little recognition and emphasis. Therefore, it is important to know the effect of different levels of saliva on the caries process and whether this effect is dose-dependent.

Several variables related to the biochemical composition act additively to explain the anticaries properties of saliva. One of the most relevant caries-protective factors in saliva is the buffer capacity, given mainly by bicarbonate (HCO_3_^−^). The bicarbonate system stands out because when an acid is added to the oral environment, it reacts releasing a weak carbonic acid that rapidly dissociates into water and CO_2_, resulting in a complete elimination and increasing the pH of the oral environment, following the chemical balance: H^+^ + HCO_3_^−^ ⇋ H_2_CO_3_ ⇋ H_2_O + CO_2_ [7]. The phosphate and the protein buffers are also important to enhance the buffer capacity in saliva. Another protective factor found in saliva is carbonic anhydrase, which maintains pH homeostasis [8]. The salivary buffer capacity has been claimed as a protective factor in caries for a long time and is used to assess caries risk. Studies carried out to test the relationship between salivary buffer capacity and caries have shown an inverse relationship with root caries [9], when controlling for the presence of certain bacterial species [10].

On the other hand, when using biological experimental caries models with oral bacterial biofilms, there is not a clear threshold on how much saliva is necessary to mimic the oral physiological threshold for the caries-protective effect of human saliva.

Enamel and dentin net demineralization in caries is the result of frequent cycles of low pH induced by acids produced by the dental biofilm [11]. The extent of the demineralization will depend on the time required for the pH to return to neutral values, which is mainly controlled by the amount and composition of saliva [12]. Hence, saliva has been described as one of the most relevant modulatory factors of the caries process [13]. When saliva exposure to dental tissues is restricted, pH drop is more accentuated and the recovery period is longer than when normal salivary flow exposure is allowed [14]. Hyposalivation can lead to decreased pH regulation and buffer capacity, as well as altered calcium–phosphate homeostasis, bacterial metabolism, and clearance of debris and food particles [15]. Given the key role of saliva in the caries process, in vitro models where this variable is not considered would lack clinical relevance. In vitro models typically use saliva only to form the acquired pellicle [16], but very few have used human saliva as part of the culture medium [17]. Hence, there is little evidence regarding the anticaries effect of saliva when used on in vitro caries models.

We aimed, therefore, to determine whether the presence of saliva in an in vitro caries model modulates the cariogenicity of sucrose. The latter was assessed through enamel and dentin demineralization, and on the properties of *Streptococcus mutans* (*S. mutans*) biofilms, using a biological caries model.

## 2. Materials and Methods

**Figure 1:**
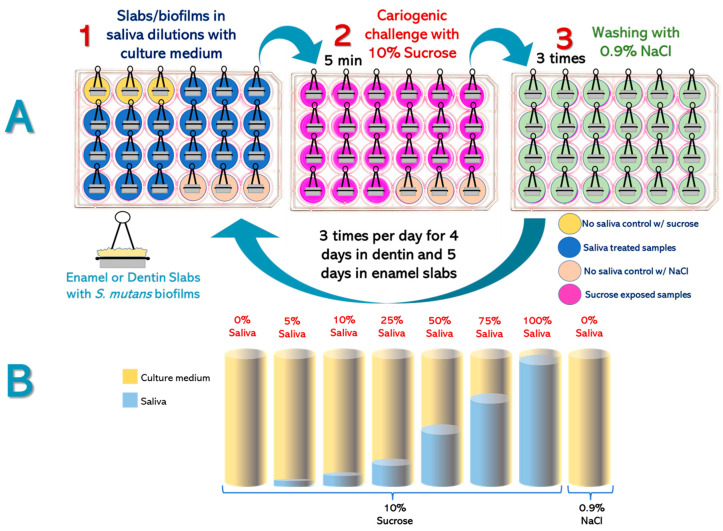
Experimental design. Panel (**A**): (1). Triplicate enamel or dentin slabs with *S. mutans* biofilms were grown into 24-well plates in trypticase soy broth (TSB) culture medium with or without saliva. (2). Slabs/biofilms were exposed to a cariogenic challenge with 10% sucrose for 5 min, except the negative caries control, exposed to 0.9% Sodium chloride (NaCl). (3). All slabs were washed 3 times in wells filled with 0.9% NaCl and then returned to the growth media. Media were changed twice per day, at 9:00 AM and 5:00 PM, during the 4 day-period for dentin, and 5 days for enamel slabs. Panel (**B**): Representation of the eight tested groups based on saliva content (0 to 100%) and cariogenic challenge (10% sucrose) or 0.9% NaCl.

Experimental Design (Figure 1). Enamel and root dentin bovine slabs (n = 24, per substrate) were grown using a standardized biological caries model using *S. mutans* biofilms (Figure 1A). Slabs/biofilms were divided into 8 groups, with different saliva and culture medium proportions (Figure 1B). Cariogenic challenges were carried out with 10% sucrose 3 times per day. Caries-positive and caries-negative controls were also included. Saliva-containing culture media were replaced twice per day and the pH was measured after each change, as an indicator of the biofilm’s acidogenicity [18]. The entire experiment was independently repeated, with each treatment in triplicate (n = 6). After every experimental phase (5 days for enamel and 4 days for dentin), surface microhardness loss (demineralization), biomass, viable *S. mutans* cells, and insoluble extracellular polysaccharides (IEPS) were evaluated.

Enamel and dentin slabs preparation. Caries- and calculus-free bovine incisors were used. Teeth were disinfected with sodium hypochlorite (NaOCl 5%) and stored in distilled water, for no more than 30 days. Twenty-four enamel and twenty-four root dentin slabs of 7 × 4 × 1 mm dimensions were prepared using a diamond saw (LECO VC50, St. Joseph, MI, USA). Slabs were initially polished with an automatic polisher (LECO SS200) and finished with a series of Sof-Lex discs (3M, St. Paul, MN, USA) (Supplementary Material Figures S1 and S2).

The Knoop surface microhardness of each slab was determined, as previously described [16]. To avoid variability derived from the dental tissues, excess slabs were prepared and only those within a surface microhardness of 352.56 ± 1.6 kg/mm^2^ for enamel and 55.37 ± 1.7 kg/mm^2^ for dentin were included. Slabs were mounted in a specially designed device (made with orthodontic wires) to ensure they were firmly attached, and could be securely inserted and removed into the wells. Slabs were sterilized with ethylene oxide and randomly allocated into a treatment well of a 24-well plate (Costar, Corning, NY, USA). Slabs were hydrated with sterile distilled water before the beginning of the experiments.

Saliva collection and processing. Three volunteers donated whole stimulated saliva for all the experiments. Volunteers met the following inclusion criteria: age range from 18 to 30 years old, normal salivary flow according to the ranges indicated in the Cariogram software [19], systemically healthy, without active caries lesions, not having used any medication 3 months prior to collection, not having consumed tobacco or alcohol 48 h prior to saliva donation, not having consumed food or liquids for at least 2 h prior to collection, and not having orthodontic appliances [20]. Subjects were comfortably seated, with their head bent forward to gently pour saliva secreted for 5 min into a sterile 15 mL tube (Kima, Arzergrande, Italy) [21]. To minimize variations, saliva collection was standardized, using the same conditions for all donors [22]. Saliva collection was conducted over a one-month period during the autumn season, with average temperatures ranging from 12 °C to 29 °C. The study protocol was approved by the Ethics Committee of the University of Talca under the folio number 14-2021.

Salivary protein concentration was determined via the BCA-Pierce method, using the Pierce BCA Protein Assay Kit (Thermo Scientific, Waltham, MA, USA), and readouts were carried out in a microplate reader (TECAN, Infinite M200 Pro, Männedorf, Switzerland) at 562 nm. Saliva pH was determined using a microelectrode (Orion 910500, Thermo Scientific, Waltham, MA, USA) coupled to a digital pH meter (Orion Star A211, Thermo Scientific).

Fresh saliva samples were obtained twice per day during the entire duration of the experimental phase. Volunteers collected 20 mL of whole stimulated saliva in a period not exceeding 30 min, using paraffin films (Parafilm M; American Can Co., Neenah, WI, USA). Saliva was collected in graduated plastic 15 mL tubes, discarding the foam formed during the process. Saliva samples were pooled and mixed with the protease inhibitor phenylmethylsulfonyl fluoride (PSMF) at 1:100 (*v*/*v*) and adsorption buffer (AB) 1:1, prepared according to a previously described methodology [23]. Saliva samples were centrifuged at 3800× *g*, for 10 min at 4 °C and filtered with a 0.22 µm microfilter (Rapid Flow, Nalgene, Rochester, NY, USA).

To control excessive variations in the pooled saliva used for the experiments, both pH and total protein concentration of the donated saliva were tested among the 3 healthy donors. The mean protein concentration was 1.14 ± 0.23 (mg/mL) and the mean pH was 7.72 ± 0.31, without significant differences among donors.

Biofilm formation. From frozen stocks, inocula of *S. mutans* UA159 were prepared in brain heart broth (BHI) (Merck, Darmstadt, Germany), supplemented with 10% glucose and incubated at 37 °C and 10% CO_2_ (Panasonic, MCO-19M, Osaka, Japan) for 18 h. Ultrafiltered saliva was used to simulate the acquired pellicle on the enamel and dentin surfaces [24,25]. After the formation of the acquired pellicle, biofilms were initially formed in sugar-free trypticase soy broth (TSB) (Merck, Darmstadt, Germany), supplemented with 1% sucrose, for 8 h, at 37 °C and 10% CO_2_. A growth time of 16 h to mature the biofilms was allowed by transferring the slabs to a new plate with the same medium but supplemented with 0.1 mM glucose, which is the basal glucose concentration in saliva, for an additional 8 h up to 24 h [18].

Biofilm exposure to experimental conditions. Enamel and dentin slabs were used to grow *S. mutans* biofilms. Once mature, slabs/biofilms were divided into the following experimental groups (n = 3/per group), each consisting of saliva mixed with TSB culture medium (*v*/*v*), at 0%, 5%, 10%, 25%, 50%, 75%, and 100% saliva in TSB medium. Biofilms were challenged with 10% sucrose for 5 min 3x/day to simulate the main meals and the subsequent pH drop. The 0% saliva + 10% sucrose group and the 0% saliva + 0.9% sodium chloride (NaCl) were used as caries-positive and caries-negative controls, respectively. After each cariogenic challenge, slabs/biofilms were washed 3 times with NaCl 0.9% (*w*/*v*) and relocated into the corresponding well with culture medium and saliva. Biofilms were incubated at 37 °C and 10% CO_2_. Culture medium was changed twice per day, after the first and after the last daily cariogenic challenge with sucrose.

Dependent variables. The following dependent variables were assessed for each experimental condition, either from the medium, the biofilms, or the enamel or dentin slabs.

Biofilm acidogenicity. To reproduce the physiological pH variations in the mouth [18], a cyclical pH model was created by exposing the biofilm to cariogenic challenges with a 10% sucrose solution, 3x/day. The pH of the culture medium was measured in each well, twice per day, after each medium change and for the entire duration of the experimental phases [18]. The pH was assessed using a microelectrode (Orion™ 910.500, Thermo Scientific, Waltham, MA, USA) coupled to a digital pH meter (Orion™ Star A211, Thermo Scientific). To facilitate the interpretations and comparisons of the different pH curves obtained at the different time points, the area over the curve (AOC) was used [18], which represents all the dynamic ranges of pH, but in a single value. Using a common cut-off point allows the evaluation and comparison of pH dynamics with the other experimental groups. The AOC was calculated considering a pH 6.87 as the cut-off point, being the lowest pH value obtained by the caries-negative control with 0% saliva + 0.9% NaCl.

Enamel and dentin demineralization. The demineralization of the slabs that occurred during the cariogenic challenges was estimated by the surface microhardness (SH) loss. SH as an indicator of demineralization has been widely used [18]. SH was obtained from each enamel and dentin slab once the experimental phase was concluded. Slabs were retrieved detaching the biofilm by vortexing for 30 s in 0.9% NaCl and then washing 3 times with 0.9% NaCl. Prior to the start of the experiments, the SH of each slab was evaluated by performing 3 indentations every 100 μm, which led to an initial SH (SHi). Once the experimental phases were carried out, slabs were reassessed by creating a set of 3 additional indentations next to the initial measurements to obtain the final SH (SHf). Thus, the percentage of SH loss (%SHL) was obtained using mean values and calculated as follows: (SHi − SHf) × 100/SHi.

Biofilm analysis. Once the experimental phases were completed, the enamel and dentin slabs were washed 3 times and transferred to Eppendorf tubes (Eppendorf AG, Hamburg, Germany) with 1 mL of NaCl 0.9% (*w*/*v*). Biofilms were detached, as indicated above, and the following variables were determined from the suspension obtained.

Biomass: The crystal violet method was used with modifications [18]. An aliquot of 200 µL of biofilm formed on each slab was transferred to an Eppendorf tube, and then 600 µL of 95% ethanol was added and incubated at −20 °C for 30 min. Samples were centrifuged for 10 min at 4 °C and 10,000× *g* (Thermo Scientific, Heraeus Megafuge 16R, MA, USA). The supernatants were removed from each tube and 500 µL of 75% ethanol was added and centrifuged again. In total, 50 µL of 0.1% crystal violet dye was added to each suspension and incubated for 10 min at room temperature and centrifuged for another 10 min. The resulting pellet was washed twice with 450 µL 1X PBS. Supernatants were discarded and 200 µL of 95% ethanol was added until the biofilm was solubilized. Samples were incubated for 15 min at room temperature and 100 µL of the mixture was transferred to a 96-well microplate and read at 600 nm (Tecan, Infinite M200 Pro, Männedorf, Switzerland).

Viable *S. mutans* cells: The drop plate method was used to count viable *S. mutans* cells [18]. Briefly, 8 serial dilutions 1/10 (*v*/*v*) in 0.9% NaCl of the biofilm suspension were used. From each dilution, 3 drops (20 µL) were seeded on a Mitis Salivarius Bacitracin agar plate (MSB, BD, Sparks, NV, USA). After 48 h of incubation, at 37 °C, with 10% CO_2_, colony-forming units (CFU) were counted with the naked eye, from the dilution where the colonies could be individually counted. The results were expressed in CFU/mL of the biofilm suspension, after normalizing by the dilution and the biomass values.

Insoluble extracellular polysaccharides (IEPS): A previously described methodology was used [18]. An aliquot of 200 μL of the biofilm suspension was centrifuged at 10,000× *g* for 5 min at 4 °C. The resulting pellet was treated with 300 μL of 1 M NaOH, homogenized, and centrifuged. The pellet was treated with three volumes of 500 μL of cold 100% ethanol and incubated for 30 min, at −20 °C. Samples were centrifuged and washed 3 times with 500 μL of cold 75% ethanol and centrifuged again. The resulting pellet was resuspended in 1000 μL of 1 M NaOH. The total concentration of carbohydrates was calculated by the sulfuric phenol method [18] in a microplate reader at 490 nm. The results were normalized by biomass and expressed as µg/mL.

Statistical analysis. The means of the dependent variables were compared with the normality and equality of variance tests. All data were analyzed with the statistical software SPSS 15.0 for Windows (IBM Corporation, New York, NY, USA). Dependent variables were tested with ANOVA, followed by Bonferroni paired comparisons for the variables acidogenicity and biomass. The %SHL, viable cells, and IEPS were tested using non-parametric Kruskal–Wallis ANOVA, with subsequent comparisons by ranges. The level of significance was determined using a *p*-value lower than 0.05.

## 3. Results

**Figure 2:**
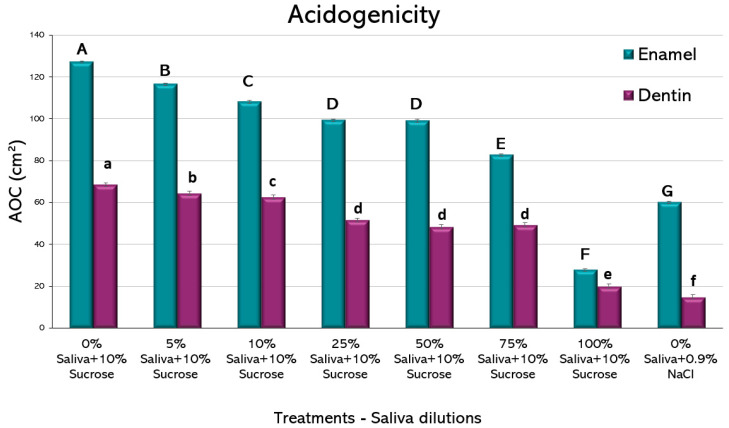
Acidogenicity induced by *S. mutans* biofilms formed on enamel and dentin slabs. Bars show mean values of the area over the curve (AOC) for each experimental condition for enamel and dentin, as indicated. Error bars represent standard deviation of the mean from 2 independent experiments, each in triplicate. Different letters represent significant differences among treatments (*p* < 0.05), uppercase for enamel and lowercase for root dentin.

All the experimental conditions showed a pH drop. When using the AOC as a single value for comparison (Figure 2), saliva-containing media showed a clear reduction in acidogenicity at all the tested dilutions. Interestingly, as little as 5% of saliva added to the culture medium showed a significant reduction in acidogenicity, both for enamel and dentin. For biofilms formed on both dental tissues, there was a dose-dependent reduction in acidogenicity, with a very low AOC for the wells containing 100% saliva + 10% sucrose. For enamel, 100% saliva + 10% sucrose was even lower than the negative control containing 0% saliva + 0.9% NaCl. The largest improvement in reducing acidogenicity was obtained from 75% to 100% saliva in both enamel and dentin groups. These findings suggest a potent function of saliva on regulating the pH of the oral environment.

**Figure 3:**
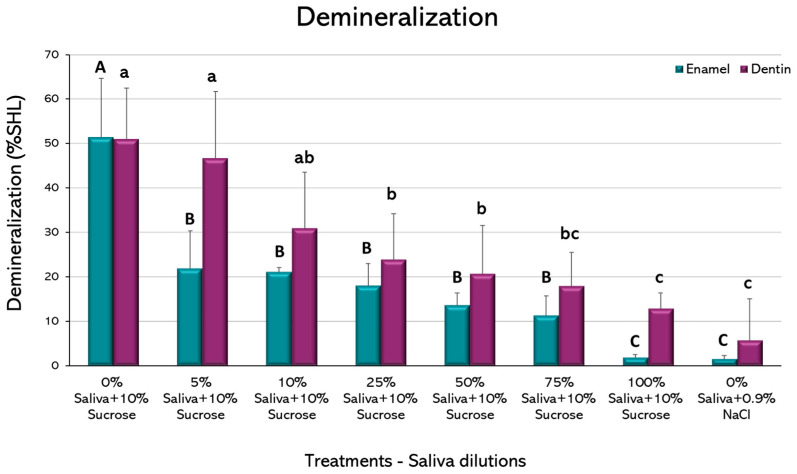
Resulting enamel and dentin demineralization by the cariogenic challenge with 10% sucrose, with different saliva dilutions. Plot shows the mean percentage of surface hardness loss (%SHL) from 2 independent experiments, each in triplicate. Error bars represent the standard deviation (SD) of the mean for each experimental condition. Different letters represent significant differences among treatments (*p* < 0.05), uppercase for enamel and lowercase for root dentin.

When demineralization (%SHL) was evaluated, a dilution-dependent inhibitory effect could be observed both in enamel and dentin (Figure 3). Interestingly, as little as 5% saliva in enamel and 25% in dentin was able to significantly reduce the %SHL, when compared to the caries-positive control, without saliva (*p* < 0.05). In this case, the largest improvement in demineralization occurred from 0% to 5% saliva in dentin and from 5% to 10% in enamel. It is important to observe that no statistically significant differences were observed between 100% saliva + 10% sucrose and the caries-negative control, without sucrose, in both dental tissues, confirming the strong caries-inhibitory effect of saliva.

**Figure 4:**
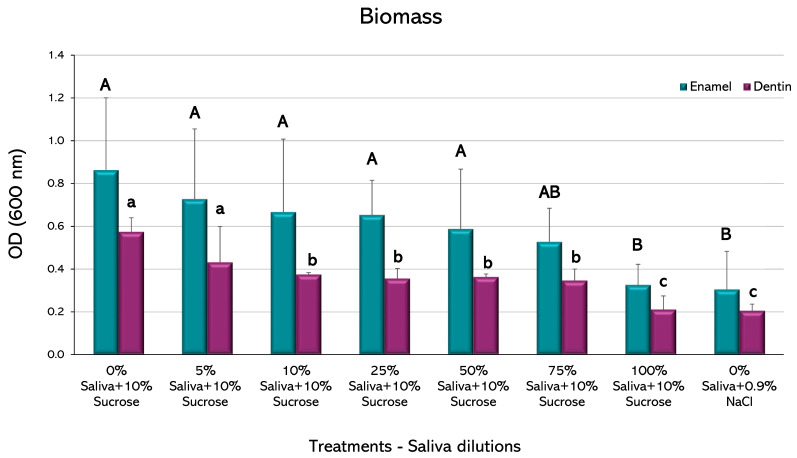
Biofilm biomass obtained from enamel and dentin slabs upon 10% sucrose exposure with varying saliva dilutions. Plot shows the mean percentage of biofilm optical density (OD_600_) from 2 independent experiments, each in triplicate. Error bars represent the standard deviation (SD) of the mean for each experimental condition. Different letters represent significant differences among treatments (*p* < 0.05), uppercase for enamel and lowercase for root dentin.

No statistically significant differences were observed in the amount of biomass among the different experimental conditions in the biofilms formed on enamel, except for the highest saliva proportion (100%), which showed a decrease compared to the caries-positive control (*p* < 0.05) (Figure 4). This group was not different to the negative control. The biofilms formed on dentin, however, showed a decrease in biomass from the 10% saliva dilution, with an even greater decrease in the 100% proportion (*p* < 0.05), which was also not different to the negative control.

**Figure 5:**
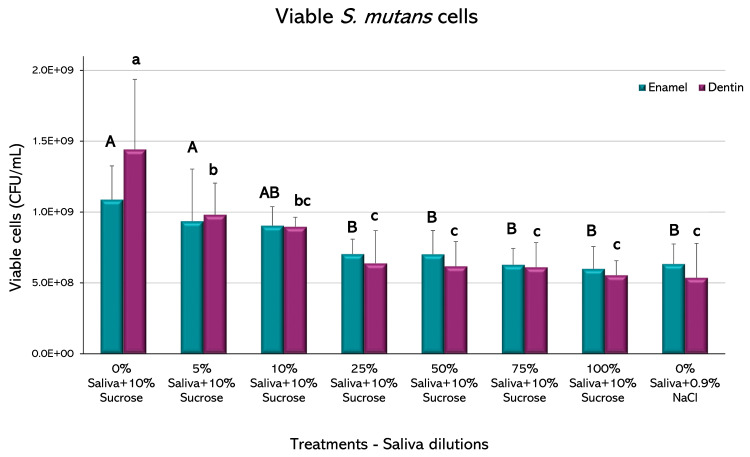
Viable *S. mutans* cells recovered from biofilms after the cariogenic challenge in the presence of saliva. Plot shows the mean percentage of colony forming units per mL (CFU/mL) from 2 independent experiments, each in triplicate. Error bars represent the standard error (SE) of the mean for each experimental condition. Different letters represent significant differences among treatments (*p* < 0.05), uppercase for enamel and lowercase for root dentin.

The biofilms recovered from the enamel showed a statistically significant decrease in the viable cells of *S. mutans* in saliva from the 25% dilution, with respect to the caries-positive control (*p* < 0.05) (Figure 5). Higher saliva dilutions did not show a significant decrease in CFU among them and with respect to the negative caries control (*p* > 0.05). Biofilms retrieved from dentin slabs had similar behavior, but the decrease in viable cells occurred from the 5% dilution.

**Figure 6:**
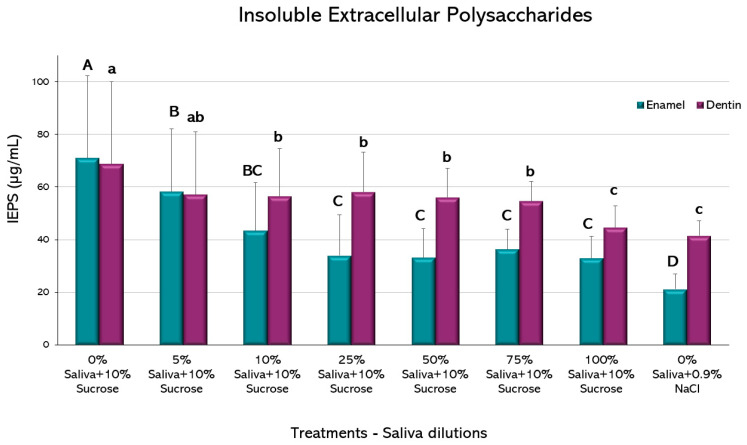
Insoluble Extracellular Polysaccharides produced by the *S. mutans* biofilms in response to 10% sucrose exposure with varying saliva dilutions. Plot shows the mean percentage of insoluble extracellular polysaccharides (IEPS) formed (µg/mL) from 2 independent experiments, each in triplicate. Error bars represent the standard deviation (SD) of the mean for each experimental condition. Different letters represent significant differences among treatments (*p* < 0.05), uppercase for enamel and lowercase for root dentin.

Compared to the caries positive control, biofilms grown on enamel had a decrease in IEPS formation from the 5% saliva (*p* < 0.05) (Figure 6). A further decrease is observed from the 25% dilution up to the 100% saliva-containing condition, but the negative control exhibited an even lower IEPS formation (*p* < 0.05).

A similar trend was observed in biofilms formed on dentin, with reductions in IEPS formation from the 10% dilution (*p* < 0.05). Further dilutions were not different to the 10%, except for the 100% saliva that resulted in lower IEPS formation, without difference to the negative control (*p* > 0.05).

## 4. Discussion

Multiple reports have described the protective role of saliva in oral health. Indeed, a severe decrease under certain systemic conditions or due to polypharmacy leads to a detrimental oral condition, with a particular impact on the onset of caries lesions.

Although decreased salivary flow has been clearly found to be associated with increased caries prevalence [12,15], we aimed at establishing a quantitative threshold at which saliva can exert its anticaries effect in an in vitro caries biological model. We observed that little amounts of saliva in the simulated oral environment could significantly reduce dental demineralization and positively modify the properties of a cariogenic bacterial biofilm. In other words, under the conditions tested, the presence of saliva in the culture medium reduced or even completely masked the deleterious effects of sucrose. The dilution-dependent effects found in this study suggest that although minimal amounts of saliva can start hampering the cariogenic effect of sucrose on demineralization and the properties of the biofilms, the presence and the amount of saliva in the culture media are important in order to modulate the cariogenic potential of tested products when using caries models.

The amount of saliva seems to play a role in enhancing the pH-controlling properties. Indeed, only a mild effect could be observed for dilutions lower than 25% of saliva in the medium (Figure 2). Hence, people suffering from xerostomia with hyposialia derived from systemic diseases, or from the use of several medications, are at higher caries risk due to the lack of enough caries protective factors being contained in saliva.

Regarding the effect of salivary proteins in caries, a systematic review concluded that there was not sufficient evidence to establish salivary proteins as biomarkers for caries [26]. Specific salivary proteins, however, may have an anticaries effect derived from antibacterial activity, including the peroxidase systems, lysozyme, lactoferrin, and histatins. Antimicrobial peptides (AMPs) belonging to the innate immune system are also present in saliva [27]. Some of the best characterized antimicrobial peptides are α- and β-defensins, human cathelicidin LL-37, and histatins [28]. A recent study that aimed to assess the levels of salivary antimicrobial peptides of children without carious lesions and with early childhood caries (ECC) failed to find differences in the levels of antimicrobial peptides [29], although, in other studies, LL37 levels have been associated with a lower caries incidence in children [30]. Conversely, we have shown that antibodies, particularly IgA, are differentially expressed in saliva from caries-free subjects, suggesting an additional role for adaptive immunity in caries [31]. Saliva has been proposed to be part of the innate immunity in caries [32]. Further studies should deepen the anticaries effect of saliva with different amounts of antimicrobial peptides.

In our biological model using saliva, there were differences between enamel and dentin. Enamel demineralization was strongly reduced by the presence of minimal amounts of saliva in the media, even as low as 5% (Figure 3). In dentin, higher proportions (25%) were needed to counteract the effect of 10% sucrose in the medium, when compared to the caries-positive control. This is important, as it could provide an explanation for the increased susceptibility of older adults to develop root caries lesions rather than coronal caries lesions. [33]. Enamel and dentin are differentially affected by the acidic challenge [34], where enamel partially losses surface minerals leading to softening and subsequent roughening. Conversely, root dentin can be equally demineralized, but invaded by bacteria from the dental biofilm as well [35]. The much higher organic content of dentin allows for collagen demineralization and denaturation, which makes it more demanding for the protective substances in saliva to counteract. A more porous structure is also expected in dentin, which allows the retention of acids and *S. mutans* cells, increasing the mineral loss. The differential demineralization of both tissues has been widely demonstrated in most in vitro studies.

Biofilms formed on enamel only reduced the total biomass when saliva was at 100%, whereas biomass reduction occurred at a 10% dilution in dentin (Figure 4). It is reasonable to speculate that this difference could be due to the more organic nature of dentin, but the exact mechanism is unknown and deserves further research for its possible practical implications. On the other hand, small fractions of saliva were enough to reduce viable microorganisms on both tissues (Figure 5). Antibacterial peptides contained in saliva have been described as potent bacterial inhibitors [36]; so, viable cells from the biofilms may be targets for salivary proteins, presenting a bacteriostatic rather than bactericidal effect. Another essential part of the biofilm is the polysaccharide fraction, which makes up a significant amount of the entire biomass. Our results showed that saliva dilutions over 10% were capable of reducing the amount of IEPS from the biofilms formed on enamel and dentin (Figure 6). IEPS derive from sugars and constitute the scaffold where bacteria adhere and proliferate, leading to mature biofilms [37,38]. The fact that all treatment groups in the current study had the same amount of sucrose strongly suggests that some salivary component may inhibit EPS synthesis. Although a relation between saliva and IEPS production has been scarcely described, evidence suggests that the quality and composition of saliva can module the virulence and IEPS expression on the biofilms [39]. Indeed, it has been described that the lactoperoxidase system present in saliva inhibits the glycosyltransferases of *S. mutans* in vitro [40], which are the *S. mutans* enzymes responsible for synthesizing IEPS.

Although the use of human fresh saliva during the whole experiment added clinical relevance to this model, we acknowledge the intrinsic limitations of an in vitro approach. Besides saliva and its components, the clinical oral environment has several other cariogenic and inhibitory variables taking part in the final caries outcome. Thus, diet, hygiene habits, hard tissue composition and morphology, differential fluoride exposure, among many others are not included in the model and impair the generalizability of the results. An important limitation is the monospecies nature of the tested biofilms. Although we are well aware that the dental biofilm comprises hundreds of coexisting species [41], our experimental design was meant to test the hypothesis that certain quantitative salivary thresholds are needed to verify the effect of the fluid in reducing the cariogenicity of sucrose. If we had included a greater number of species in the biofilms, the model would have been subject to uncontrollable variability, potentially resulting in ambiguous or inconclusive results. We decided to use stimulated saliva, as it provides higher amounts of fluid to be used in the experiments in a shorter time. From a biological point of view, however, unstimulated saliva could originate different and more meaningful results. Stimulated and unstimulated saliva have different origins from different salivary glands [42]. It would be of interest to repeat these experiments to verify whether both types of saliva result in different anticaries effects. Finally, we focused on the quantitative aspects (volume of saliva), but further research is being conducted to elucidate molecular specific aspects of the protective effect in caries, and how individual variations condition the properties and composition of saliva. This would eventually demonstrate whether saliva from individuals without caries and those with active caries possess distinct anti-caries properties and could potentially facilitate the development of strategies to enhance salivary composition in high-risk individuals.

## 5. Conclusions

Our data showed that saliva has an important anticariogenic effect. The inhibitory effect of saliva on hard tissue demineralization and on the characteristics of the biofilms formed on enamel and dentin showed a clear dilution-dependent effect, and small amounts were sufficient for improvement in some of the measured features. Our results also suggest that the presence and volume of saliva are important when developing in vitro caries biological models.

## Data Availability

All data generated or analyzed during this study are included in this article. Further enquiries for specific data can be directed at the corresponding authors.

## Supplementary Materials

The following supporting information can be downloaded at https://www.mdpi.com/article/10.3390/microorganisms11061426/s1, Figure S1: Enamel and dentin slab preparation, Figure S2: Slabs mounting in the orthodontic appliance and culturing with *S. mutans*.
