# Supplementary material for: Saliva Decreases Sucrose-Induced Cariogenicity in an Experimental Biological Caries Model
Source: Microorganisms. 2023 May 29;11(6):1426. doi: 10.3390/microorganisms11061426 (PMC10304388; doi:10.3390/microorganisms11061426)
Supplement: Supplementary file 1 [file microorganisms-11-01426-s001.zip › microorganisms-2388924-supplementary.pdf]

Supplementary Material

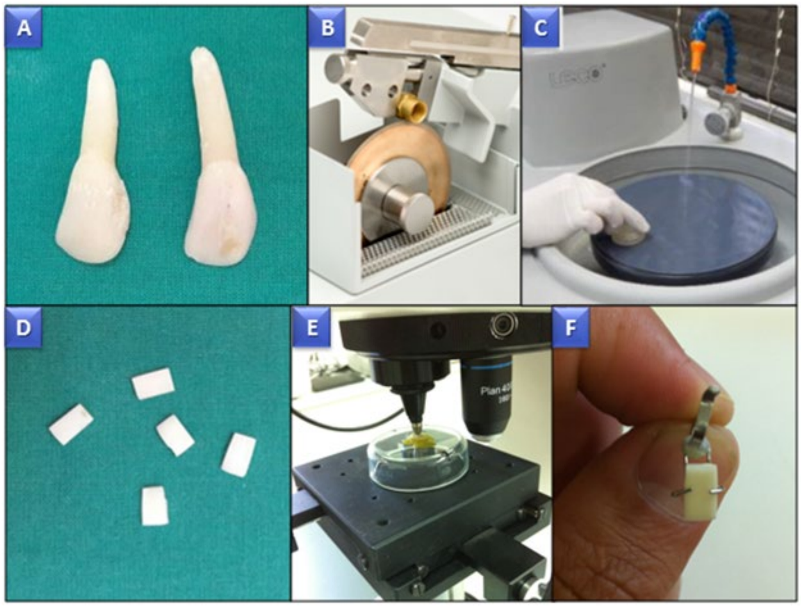

Figure S1. Enamel and dentin slab preparation. A. Selected bovine incisors. B. Diamond saw used to cut bovine incisors. C. Polishing a slab in the automatic polisher. D. Dimensioned bovine enamel slabs. E. Measurement of the initial Knoop surface microhardness with the microindenter. F. Mounting an enamel slab in the holder device, secured with composite resin.

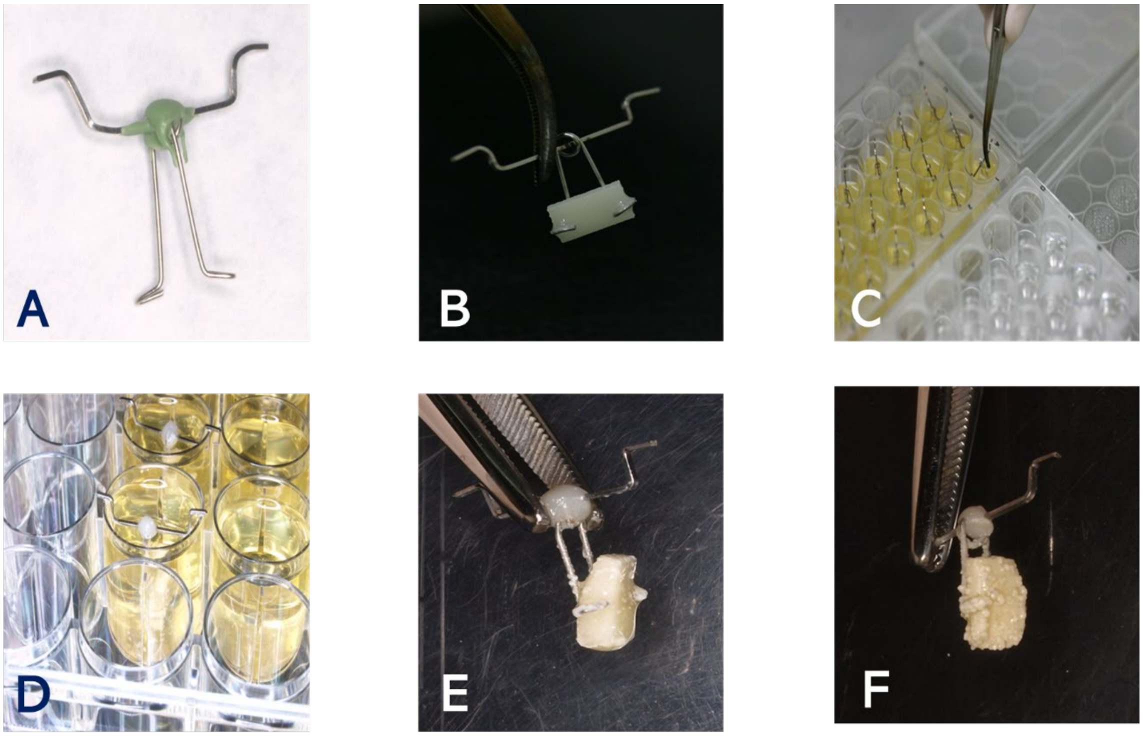

Figure S2. Slabs mounting in the orthodontic appliance and culturing with *S. mutans*. A. Orthodontic appliance to hold the slabs. B. Enamel slab mounted in the appliance. C. Slab immersion into the culture medium with *S. mutans*. D. Slab in place with culture medium during biofilm formation. E. Initial biofilm formation on the slab. F. Final aspect of the slab with the mature biofilm after 5 days.
